# Endovascular Thrombolysis in Hypothenar Hammer Syndrome: A Systematic Review

**DOI:** 10.3389/fcvm.2021.745776

**Published:** 2021-12-15

**Authors:** Philipp Jud, Gudrun Pregartner, Andrea Berghold, Peter Rief, Viktoria Muster, Katharina Gütl, Marianne Brodmann, Franz Hafner

**Affiliations:** ^1^Division of Angiology, Department of Internal Medicine, Medical University of Graz, Graz, Austria; ^2^Institute for Medical Informatics, Statistics and Documentation, Medical University of Graz, Graz, Austria

**Keywords:** thrombolytic therapy, vascular disease, arterial occlusive diseases, hypothenar hammer syndrome (HHS), endovascular treatment

## Abstract

**Objectives:** Hypothenar hammer syndrome (HHS) is a rare vascular disease caused by blunt trauma of the hypothenar region. The optimal therapeutic strategy remains debatably since no large comparative studies are available yet. We want to evaluate the effectiveness of intra-arterial thrombolysis on angiographic and clinical outcome parameters in patients with HHS by performing a systematic review of the existing literature.

**Methods:** A literature search of PUBMED/MEDLINE and SCIENCE DIRECT databases was performed up to May 2021.

**Results:** In total, 16 manuscripts with 43 patients were included in the systematic review. Intra-arterial thrombolysis led to angiographic improvement in 29 patients (67.4%) and to clinical improvement in 34 patients (79.1%). Deterioration of arterial perfusion or clinical symptoms after thrombolysis were absent. Post-interventional complications were reported in only one patient (2.3%) without any bleeding complication. Logistic regression analyses demonstrated that a combined administration of fibrinolytics and heparin was associated with a significantly improved arterial patency [OR 12.57 (95% CI 2.48–97.8), *p* = 0.005] without significant amelioration of clinical symptoms [OR 3.20 (95% CI 0.6–18.9), *p* = 0.172]. The use of rt-PA compared to other fibrinolytics and a prolonged thrombolysis duration of more than 24 h did not show statistically significant effects. Intra-arterial thrombolysis was significantly less effective in patients who had undergone thrombolysis with a delay of more than 30 days regarding clinical improvement [OR 0.07 (95% CI 0.00–0.54), *p* = 0.024].

**Conclusions:** Intra-arterial thrombolysis with a combination of fibrinolytics and heparin is an effective and safe therapeutic option in patients with acute HHS.

## Introduction

Hypothenar hammer syndrome (HHS) represents a rare ischemic disease of the hand and digital arteries which is typically caused by mechanical trauma to the distal ulnar artery at the hypothenar region (1–3). Ulnar artery branches into two segments after exiting Guyon's canal to form the deep and superficial palmar arch. As the superficial branch of the ulnar artery passes the hypothenar muscles proximately the hook of hamate and pisiform bone, repetitive blunt trauma of the hypothenar region may cause local thrombus or aneurysm formation due to short but intensive mechanical compression of the ulnar artery toward the pisiform and hamate bones. As a consequence, distal thromboembolism of the digital arteries may occur. Depending on the predominant ulnar arterial pathology, HHS can be classified into a thrombotic and an aneurysmal form while both types are frequently accompanied by distal digital embolization (4, 5). Von Rosen firstly described in 1934 a factory worker with thrombosis of the ulnar artery after repetitive blunt trauma of the hand (6). In 1970, Conn et al. named this entity as HHS (1). Occupational strain to the hollow of hand is associated with an increased risk to develop HHS, especially after using the hand as a hammer or exposure to vibrating tools (7). Previous studies reported an increased incidence among mechanics, metal workers or smiths, however, HHS has also been recognized among athletes or truck drivers (3, 8–11). Overall, the prevalence rate of HHS is about 1% in patients exhibiting Raynaud's phenomenon (9).

Symptoms of HHS vary from subtle digital discoloration and cold intolerance to severe digital and hand ischemia with threatening digital gangrene. Diagnostics in HHS seem to be well-established including physical status with Allen's test, digital acral plethysmography, color-coded ultrasound and angiography. Furthermore, laboratory testing may be considered to exclude potential immunological or hematological diseases, especially in atypical presentations of HHS (3). Compared to well-established diagnostics, current treatment in HHS is often empiric due to absent randomized trials or large comparative studies and due to a wide spectrum of symptoms and disease severity. Treatment options include symptom-oriented medical treatment, like vasodilators, antiplatelet therapy and analgesics, risk factor management, including tobacco cessation or hand protection in less severe cases, as well as invasive options, like thrombectomy, bypass surgery and intra-arterial thrombolysis especially in acute and subacute hand ischemia and aneurysmal changes (3). Intra-arterial thrombolysis is increasingly accepted as revascularization modality for acute and subacute lower limb ischemia (12). However, its role in upper limb ischemia, especially in HHS, remains unclear. Although a review reported comparable clinical results regarding limb salvage rates in acute upper limb ischemia between surgery and intra-arterial thrombolysis supporting the utility of intra-arterial thrombolysis also for distal ischemia of the upper limb, there are only a few case reports and case series reporting treatment with intra-arterial thrombolysis in HHS (13).

The aim of this study was to evaluate the effectiveness of intra-arterial thrombolysis on clinical and angiographic outcome parameters in patients with diagnosed HHS by performing a systematic review of the scientific literature on this topic.

## Materials and Methods

A systematic search of the literature was performed to evaluate in adults with HHS the effectiveness of intra-arterial thrombolysis on clinical and angiographic outcome parameters qualitatively and quantitatively. Primary endpoints were changes of clinical symptoms and thrombotic depositions after intra-arterial thrombolysis. Secondary endpoints were the occurrence of post-interventional complications, the effectiveness of a combined administration with fibrinolytics and heparin, as well as the influence of different fibrinolytic agents, duration of intra-arterial thrombolysis and of patients' delay to presentation on clinical and angiographic outcome. The systematic review was performed in accordance with the PRISMA 2009 guidelines while no review protocol was prepared (14). We pre-specified the following inclusion criteria: (a) the study had to report intra-arterial thrombolysis in patients with diagnosed HHS, (b) reported patients had to be 18 years or older; (c) the study had to report relevant outcome data regarding either angiographic or clinical changes after thrombolysis.

An electronic literature search of the databases PUBMED/MEDLINE and SCIENCE DIRECT was conducted in May 2021. The keywords “hypothenar hammer syndrome” together with “thrombolysis,” “fibrinolysis,” “fibrinolytics,” “urokinase,” “streptokinase,” “alteplase,” “tenecteplase,” “reteplase,” or “rt-PA” were used. We considered publications in English, French, and German, screened all abstracts and retrieved the full printed manuscript of all abstracts with a possible reported use of thrombolytic therapy in HHS. Additionally, full printed papers were retrieved when abstracts were missing and from studies with a possible reported use of thrombolytic therapy in HHS, which were not detected by the search in PUBMED/MEDLINE and SCIENCE DIRECT but cited in one of the retrieved papers. All abstracts and full texts were evaluated independently by two investigators (P.J. and F.H.). Data were extracted from all eligible publications regarding patient characteristics, including age, profession or initial symptoms, endovascular specifications, including thrombolysis duration or used fibrinolytics, as well as clinical and angiographic outcome parameters. Clinical and angiographic changes after intra-arterial thrombolysis were qualitatively assessed into “complete relief of symptoms” or “complete arterial patency,” “improvement with mild symptoms” or “improvement with residual thrombotic depositions,” “no change of symptoms” or “no change of arterial patency,” and “deterioration of symptoms” or “deterioration of arterial perfusion.” Additionally, quantitatively assessed clinical and angiographic changes were recorded if assessment methods to quantify clinical or angiographic changes were reported in the retrieved manuscripts. Furthermore, numbers of affected hand and digital arteries with completely restored patency without thrombotic depositions after intra-arterial thrombolysis were recorded. All eligible publications were evaluated by a proposed tool for methodological quality assessment of case reports and case series using an aggregated quality score (15).

### Ethical Approval and Informed Consent

The study was approved by the institutional review board (EK 28-260 ex 15/16) and all research was carried out according to the relevant guidelines and regulations.

### Statistical Analyses

Categorical parameters are presented as absolute and relative frequencies whereas for continuous parameters mean and standard deviations (SD) or median as well as range (minimum to maximum) are given. Since this systematic review consists of case reports and case series, individual patient data are available for most parameters. Therefore, simple logistic regression analyses to assess the influence of several risk factors regarding clinical and angiographic outcome was performed as univariable analysis. Clinical and angiographic outcome parameters were trichotomized into “any improvement,” “no change” and “any deterioration.” Results are presented as odds ratios (OR) and 95% confidence intervals (CI). Statistical significance was assumed for *p*-values < 0.05. The statistical analyses were performed using R version 3.6.1.

## Results

**Figure 1:**
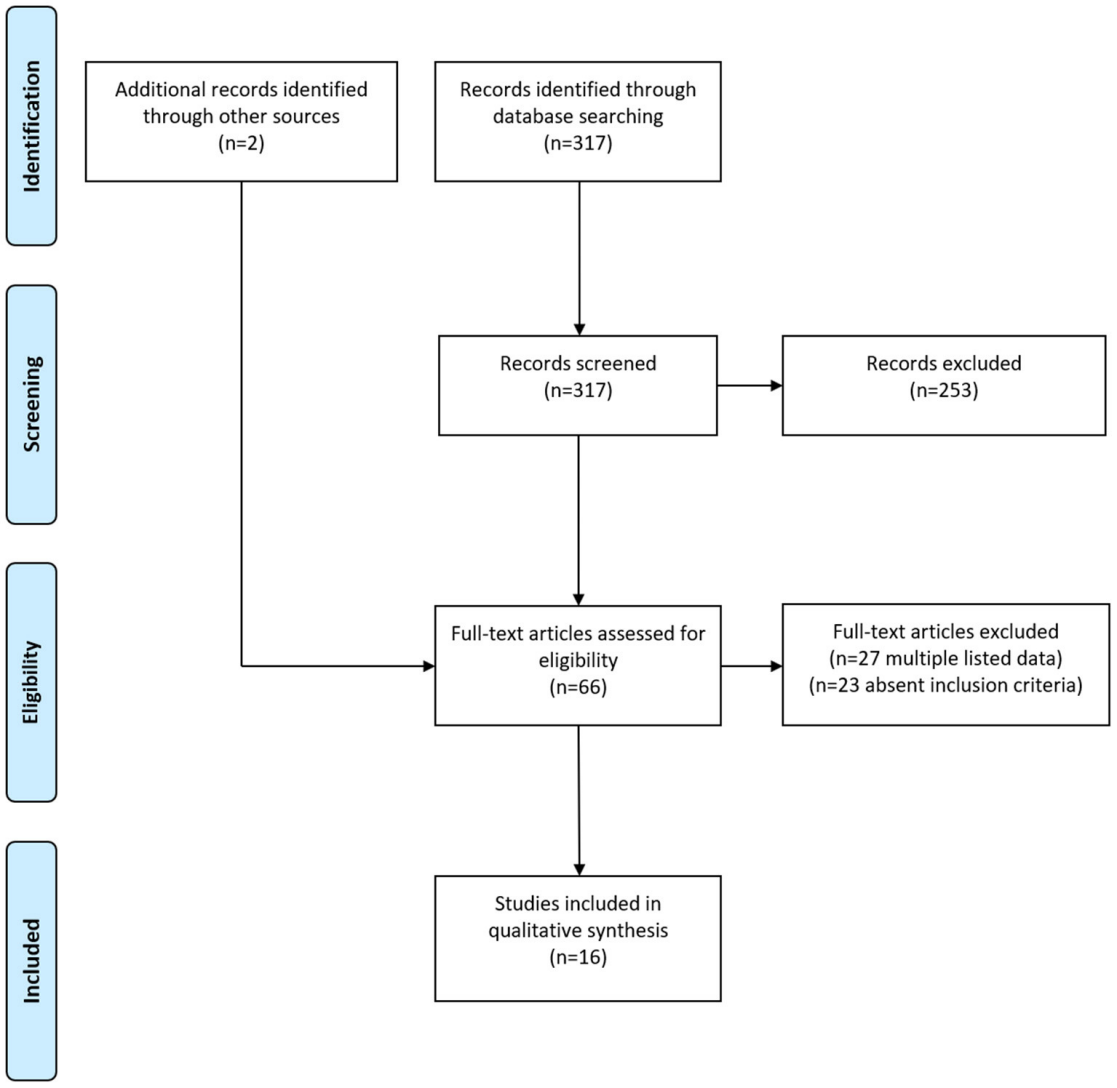
Flow diagram illustrating study identification process.

In total, 317 primary publications could be identified by the literature search using the matching keywords. Abstracts were screened for a possible reported usage of thrombolytic therapy in patients with HHS and full-texts of 64 papers were retrieved. After removing duplicates listed in both PUBMED/MEDLINE and SCIENCE DIRECT and publications that did not report the usage of a thrombolytic therapy, 14 eligible publications could finally be identified (10, 11, 16–27). Additionally, we included two further studies with a reported use of thrombolytic therapy in HHS in our systematic review which were not detected by the literature search in PUBMED/MEDLINE and SCIENCE DIRECT, but which were cited in one of the retrieved full-text manuscripts (28, 29). The flow diagram of the selection process for this systematic review is shown in Figure 1. Thus, overall 16 papers published between 1976 and 2021 were included in our systematic review. Ten publications (62.5%) were case reports and six (37.5%) were case series, respectively. The studies had a median quality score of 6 points (range 4–8) out of a maximum of 8. Detailed information about the included papers are provided in Table 1; Supplementary Table 1.

**Table 1:** Detailed results of the assessment of the methodological quality of the individual paper regarding Murad et al. (15).

**Author**	**Selection**	**Ascertainment**	**Causality**	**Reporting**	**Overall score**
Bakhach et al. (16)	1	2	3	1	7
Biskup et al. (17)	1	2	2	0	5
Capek et al. (18)	1	2	4	1	8
Friedrich et al. (19)	1	1	3	1	6
Jud et al. (20)	1	2	4	1	8
Kartchner et al. (28)	1	1	3	1	6
Lawhorne et al. (21)	1	2	2	1	6
Müller-Mai et al. (22)	1	1	2	0	4
Pfyffer et al. (29)	1	1	4	1	7
Pineda et al. (23)	1	2	3	0	6
Schneider et al. (24)	1	2	2	1	6
Shukla et al. (11)	1	1	2	1	5
Wheatley et al. (25)	1	2	2	1	6
Wörnle et al. (26)	1	2	3	1	7
Yakubov et al. (27)	1	2	2	1	5
Zayed et al. (10)	1	2	3	1	7

Within the 16 papers included in this review, 43 patients (34 male, 1 female, 8 unknown) were described who underwent intra-arterial thrombolysis due to HHS. Mean (SD) age was 47.7 (13.3) years (range 18–69 years) for 35 patients in this systematic review, while one study with eight patients reported only a mean age of 52 years for a larger study collective (29). All patients suffered from mechanical trauma to the hand. Detailed information about patients' clinical characteristics are listed in Table 2. Intra-arterial thrombolysis was performed with a median delay of 14 days (range 0.2–180 days), while the delay was unmentioned in nine cases. Thrombotic depositions of the ulnar artery, palmar arches and/or digital arteries could be observed in all patients before intra-arterial thrombolysis. Additionally, thrombotic depositions of the radial artery were described in three patients. Data about patients' interventional characteristics are listed in Table 3. One study of pooled data investigating intra-arterial thrombolysis of acute and subacute forearm, hand, and digital arterial thrombosis including eight patients with HHS reported only an access site regarding the anatomic relationship of the thrombotic deposition without discrimination of the exact location (29). Therefore, we assumed a combined access site of the cubital and/or ulnar artery in those patients. Additionally, another study reported a catheter position initially in the distal radial artery which had been changed into the brachial artery due to vasospasm of the radial artery and then into the ulnar artery (19). Furthermore, two studies reported only a catheter position proximal to the occlusion without discrimination of the exact location (19). According to their documented arterial occlusions, we assumed a combined catheter position of the brachial and/or ulnar artery in 15 cases (34.9%).

**Table 2:** Patients' clinical characteristics before intra-arterial thrombolysis.

**Clinical characteristics**	**Total (*n* = 43)**
Male, *n* (%)	34 (79.1)
Female, *n* (%)	1 (2.3)
Unknown, *n* (%)	8 (18.6)
Age in years^*^
Mean ± SD	47.7 ± 13.3
Range minimum-maximum	18–69
Occupational mechanic trauma, *n* (%)	43 (100)
Occupation	
Farmer	4 (9.3)
Construction worker	5 (11.6)
Metal worker	2 (4.7)
Electrician	1 (2.3)
Truck driver	2 (4.7)
Wood worker	1 (2.3)
Carpenter	4 (9.3)
Janitor	1 (2.3)
Mechanic	2 (4.7)
Laborer	1 (2.3)
Furniture mover	1 (2.3)
Orthopedist	1 (2.3)
Tennis player	1 (2.3)
Hockey player	1 (2.3)
Vibration trauma	1 (2.3)
Trauma after using the hand as a hammer	3 (7.0)
Unknown	12 (27.9)
Morphologic subtype of HHS, *n* (%)	43 (100)
Aneurysmal	6 (14.0)
Non-aneurysmal	17 (39.5)
Unknown	20 (46.5)
Affected hand, *n* (%)	43 (100)
Right hand	16 (37.2)
Left hand	7 (16.3)
Both hands	0 (0)
Unknown	20 (46.5)
Clinically affected fingers, *n* (%)^†^
1st finger	1 (2.3)
2nd finger	12 (27.9)
3rd finger	15 (34.9)
4th finger	24 (55.8)
5th finger	15 (34.9)
Symptoms, *n* (%)^†^
Pain	29 (67.4)
Pallor	18 (41.9)
Cold intolerance	12 (27.9)
Paresthesia	18 (41.9)
Cyanosis	15 (34.9)
Necrosis	5 (11.6)

*HHS, hypothenar hammer syndrome; SD, standard deviation*.

* *Not including eight patients from one study (29) providing only mean age and range for a larger study collective*.

† *More than one listed option possible*.

**Table 3:** Patients' interventional characteristics.

**Interventional characteristics**	**Total (*n* = 43)**
Thrombotic deposition before thrombolysis, *n* (%)^†^
Ulnar artery	31 (72.1)
Radial artery	3 (7.0)
Palmar arches	15 (34.9)
Digital arteries	41 (95.4)
Access site, *n* (%)
Brachial	15 (34.9)
Femoral	14 (32.6)
Radial	1 (2.3)
Combined (brachial/ulnar)	8 (18.6)
Unknown	5 (11.6)
Sheath size, *n* (%)	
4F	15 (34.9)
5F	12 (27.9)
6F	1 (2.3)
Peripheral venous catheter	10 (23.3)
Unknown	5 (11.6)
Catheter, *n* (%)	
Reinforced Endhole-Infusion microcatheter	11 (25.6)
Pulsespray catheter	1 (2.3)
Unifuse catheter	2 (4.7)
Unnamed endhole catheter	9 (20.9)
Unnamed peripheral venous catheter	10 (23.3)
Unnamed 3F catheter	4 (9.3)
Unnamed 4F catheter	2 (4.7)
Unknown	4 (9.3)
Catheter position, *n* (%)	
Ulnar	13 (30.2)
Brachial	9 (20.9)
Radial	1 (2.3)
Combined (brachial/ulnar/radial)	16 (37.2)
Unknown	4 (9.3)
Fibrinolytic agent, *n* (%)	
Rt-PA	21 (48.8)
Urokinase	16 (37.2)
Streptokinase	3 (7.0)
Alteplase	1 (2.3)
Thrombolysin^®^	1 (2.3)
Unknown	1 (2.3)

*Rt-PA, recombinant tissue plasminogen activator*.

† *More than one listed option possible*.

Angiographic improvement of the arterial patency by intra-arterial thrombolysis was achieved in 29 patients (67.4%) and amelioration of the reported symptoms in 34 patients (79.1%). Intra-arterial thrombolysis led to completely patent digital arteries in 19 patients (44.2%), to a completely patent ulnar artery in seven patients (16.3%) and to completely patent palmar arches in one patient (2.3%), but failed to achieve patency in seven patients (16.3%). Information about arterial patency is missing for 15 patients (34.9%). Deterioration of clinical symptoms or arterial perfusion after intra-arterial thrombolysis was not reported. Quantitative assessment of clinical and angiographic changes was impossible due to absent reports of quantitative assessment methods in the respective retrieved manuscripts. Therefore, only qualitative assessment of clinical and angiographic changes could be performed. Details about patients' angiographic and clinical outcome after intra-arterial thrombolysis are listed in Table 4.

**Table 4:** Patients' angiographicand clinical outcome after intra-arterial thrombolysis.

**Outcome parameters**	**Total (*n* = 43)**
Angiographic changes after thrombolysis, *n* (%)	
Complete restored arterial patency	5 (11.6)
Improvement with residual thrombotic depositions	24 (55.8)
No change of arterial patency	10 (23.3)
Deteriorated arterial perfusion	0 (0)
Unknown	4 (9.3)
Clinical outcome after thrombolysis, *n* (%)	
Complete relief of symptoms	14 (32.6)
Improvement with mild symptoms	20 (46.5)
No change of symptoms	7 (16.3)
Deterioration of symptoms	0 (0)
Unknown	2 (4.7)
Restored patent arteries after thrombolysis, *n* (%)^†^	
Ulnar artery	7 (16.3)
Radial artery	0 (0)
Palmar arches	1 (2.3)
Digital arteries	19 (44.2)

† *More than one listed option possible*.

One patient (2.3%) developed an abscess of the puncture site, while bleeding complications were not reported after intra-arterial thrombolysis. In two patients (4.7%) with initial acral necrosis, necrotic tissue was surgically removed while in three other patients (7.0%) with acral necrosis no surgical intervention was necessary. Surgical bail-out procedure by bypass grafting was performed in two patients (4.7%) after intra-arterial thrombolysis due to absent clinical improvement. In one patient (2.3%), bypass grafting was performed due to aneurysm resection.

Combined administration of heparin was performed in 27 patients (62.8%) of which a constant infusion rate was prescribed with 500 IU/h in two patients (4.7%), with 833 IU/h in nine patients (20.9%) and with enoxaparin 0.4 ml/h in one patient (2.3%). Of the remaining 15 patients (34.9%), heparin dosage was administered aPTT-adjusted achieving a target range of 60 s 13 times while the heparin dosage was unmentioned two times. Combined administration of fibrinolytic agents and heparin was associated with a significantly improved arterial patency [OR 12.57 (95% CI 2.48–97.8), *p* = 0.005] compared to the administration of fibrinolytics alone. However, combined administration of fibrinolytic agents and heparin did not lead to a significant improvement of clinical symptoms [OR 3.20 (95% CI 0.60–18.89), *p* = 0.172].

Recombinant tissue plasminogen activator (rt-PA) was administered in 21 patients (48.8%). Thereof, one study reported only the administration of 14.7 ml rt-PA in seven patients (19); the remaining studies had a range of 10–541 mg. Urokinase was administered in 16 patients (37.2%). While one study reported only the minimum and maximum possible dosage of the administered urokinase without specific information of the exact dosage, the range of the remaining studies was 10,000–1,600,000 IU (29). Streptokinase was used in three patients (7.0%) (range 100,000–240,000 IU), 20 mg alteplase was given in one patient (2.3%), and 600,000 IU Thrombolysin^®^, a mix of activated fibrinolysin, profibrinolysin and streptokinase, also in one patient (2.3%). One study reported the use of intra-arterial thrombolysis but not the used fibrinolytic agent (18). The use of rt-PA was associated, albeit without statistical significance, with improved arterial patency [OR 2.66 (95% CI 0.56–13.52), *p* = 0.220] and clinical symptoms [OR 2.83 (95% CI 0.47–22.92), *p* = 0.270] compared to urokinase.

The median duration of intra-arterial thrombolysis was 17 h (range 2–72 h) while one study reported only a thrombolysis duration of >11 h (18). Another study reported only an overall minimum and maximum duration (0.5–72 h) of the intra-arterial thrombolysis without further discrimination (29). Thrombolysis duration of more than 24 h revealed neither an improvement of the arterial patency [OR 0.26 (95% CI 0.03–2.53), *p* = 0.222] nor of clinical symptoms [OR 0.50 (95% CI 0.05–11.49), *p* = 0.587] compared to a duration of 24 h or less. Intra-arterial thrombolysis was less effective in patients who had undergone it with a delay of more than 30 days regarding clinical improvement [OR 0.07 (95% CI 0.00–0.54), *p* = 0.024] compared to those patients who had undergone thrombolysis within 30 days. However, prolonged delay did not significantly influence angiographic improvement [OR 0.33 (95% CI 0.05–2.22), *p* = 0.244].

## Discussion

HHS is a rare vascular ischemic condition caused by blunt trauma of the hypothenar region. Due to its rarity, treatment options are empiric and decisions regarding conservative treatment, intra-arterial thrombolysis or surgery depend on ischemia severity, symptom duration and vessel morphology. Although reviews reported benefits of intra-arterial thrombolysis in digital and hand ischemia, including patients with HHS, data on intra-arterial thrombolysis have been derived mostly from case reports using different thrombolytic methods and different reported outcomes (13, 30, 31). Similarly, most data about surgical or conservative treatment approaches in HHS have been derived from retrospective studies or from small prospective case series using different surgical and medical approaches, but with favorable results (8, 32, 33). Nevertheless, large prospective or comparative studies investigating endovascular, surgical and conservative treatment options in HHS are yet missing. Wahl et al. (34) investigated in a systematic review of vascular traumatic disorders different therapeutic approaches and reported a pronounced inconsistency of data regarding those treatment options. However, those data included cases of HHS, thenar hammer syndrome and of hand-arm vibration syndrome while this systematic review included only patients with HHS. Furthermore, due to other search methods, we were able to include a higher number of patients treated by endovascular thrombolysis. This systemic review could demonstrate that intra-arterial thrombolysis is an effective therapeutic option in patients with acute HHS with a high rate of clinical and angiographic improvement after intra-arterial thrombolysis.

Discrepancy between improvement of clinical symptoms and patent arterial perfusion could be observed in this systematic review. Amelioration of clinical symptoms was established in 79.1% while an improved arterial perfusion with residual thrombotic depositions was achieved in only 67.4% after intra-arterial thrombolysis. Complete arterial patency was observed in only 11.6% whereas complete relief of symptoms was achieved in 32.6% of reported patients. This observation suggests that a decrease of thrombotic depositions, even without complete restoration of the arterial perfusion in digital and hand arteries, may be sufficient to provide adequate blood flow leading consequently to an amelioration of clinical symptoms. One possible explanation for this observation may be attributed to good collateral vessels of the hand, since distinct collateral vessels may also buffer ischemic symptoms in lower limb ischemia. Chronic collateral artery formation of the hand may contribute also to a benign prognosis in surgically and non-interventionally treated patients with HHS. Long-term results of surgical treatment in HHS reported relatively low patency rates although patients complained only mild symptoms and persisting symptoms occurred less frequently and less severely after 5-years follow-up period in patients with HHS treated conservatively (35–37). However, as long as there are no large comparative studies available, all three treatment options have their strengths and limitations in HHS depending on initial symptom severity and vessel morphology.

This systematic review demonstrated that a combined administration of fibrinolytic agents and heparin revealed significant higher angiographic patency rates but without significant improvement of symptoms. Nevertheless, a combination therapy with fibrinolytics and heparin should be preferred for treating patients with HHS due to a favorable risk-benefit ratio toward a potential improvement of clinical symptoms while the bleeding risk seem to be very low without any reported bleeding complication. Furthermore, this systematic review could demonstrate for the first time that intra-arterial thrombolysis in patients with HHS was less effective if administered with a delay of more than 30 days and that a prolonged thrombolysis duration of more than 24 h has no benefits on arterial patency or clinical outcome indicating that intra-arterial thrombolysis should be reserved for acute and subacute forms of HHS.

Limitations of this study are firstly the small number of patients, namely only 43, and secondly that only case reports and small case series could be included. However, this systematic review describes the largest cumulative patient cohort in HHS treated with intra-arterial thrombolysis yet. Another limitation is that long-term outcome data were only rarely reported and no analysis could be performed. Further limitations may be absent comparisons of intra-arterial thrombolysis to surgical and conservative treatment options as well as that clinical and angiographic outcome parameters could not be assessed by quantitative measurement methods.

In conclusion, the use of endovascular thrombolysis in patients with acute and subacute HHS with persisting symptoms of no longer than 30 days is associated with high clinical benefit, high arterial patency rate and low post-interventional complications. Overall, however, since randomized trials or at least large case-control study comparing endovascular, surgical and conservative medical treatment options in HHS are absent, current treatment in HHS is still rather empiric.

## Data Availability Statement

The original contributions presented in the study are included in the article/Supplementary Material, further inquiries can be directed to the corresponding author/s.

## Author Contributions

PJ and FH contributed to data collection, conceptualization, drafting, and reviewing of the manuscript. FH contributed to study design. GP and AB contributed to statistical analysis and reviewing of the manuscript. PR, VM, and KG contributed to drafting and reviewing of the manuscript. MB contributed to supervision of the manuscript. All authors approved the submitted version.

## Conflict of Interest

The authors declare that the research was conducted in the absence of any commercial or financial relationships that could be construed as a potential conflict of interest.

## Publisher's Note

All claims expressed in this article are solely those of the authors and do not necessarily represent those of their affiliated organizations, or those of the publisher, the editors and the reviewers. Any product that may be evaluated in this article, or claim that may be made by its manufacturer, is not guaranteed or endorsed by the publisher.

## Abbreviations

CI: confidence interval
HHS: hypothenar hammer syndrome
OR: odds ratio
Rt-PA: recombinant tissue plasminogen activator
SD: standard deviation.
