# Supplementary material for: Endovascular Thrombolysis in Hypothenar Hammer Syndrome: A Systematic Review
Source: Front Cardiovasc Med. 2021 Dec 15;8:745776. doi: 10.3389/fcvm.2021.745776 (PMC8714786; doi:10.3389/fcvm.2021.745776)
Supplement: Supplementary file 1 [file Table_1.docx]

Supplementary table 1: Characteristics about the eligible manuscripts for systematic review.

| Author | Year | Type of study | Number of patients | Patient age ‡ | Ascertainment |
| --- | --- | --- | --- | --- | --- |
| Bakhach et al. [16] | 1998 | Case report | 1 | 33 | Thrombolysis secondary to surgery |
| Biskup et al. [17] | 2018 | Case report | 1 | 30 | Primary thrombolysis |
| Capek et al. [18] | 1993 | Case report | 1 | 20 | Primary thrombolysis |
| Friedrich et al. [19] | 2009 | Case series | 7 | 58.6 | Primary thrombolysis |
| Jud et al. [20] | 2021 | Case series | 11 | 51.4 | Primary thrombolysis |
| Kartchner et al. [21] | 1976 | Case series | 1 | 29 | Primary thrombolysis of palmar and digital thrombosis |
| Lawhorne et al. [22] | 1986 | Case report | 1 | 44 | Primary thrombolysis with secondary surgical intervention |
| Müller-Mai et al. [23] | 2004 | Case report | 1 | 35 | Primary thrombolysis |
| Pfyffer et al. [24] | 1989 | Case series | 8 | 52 | Primary thrombolysis of lower arm, palmar and digital thrombosis |
| Pineda et al. [25] | 1985 | Case series | 2 | 43.5 | Primary thrombolysis |
| Schneider et al. [26] | 1999 | Case report | 1 | 35 | Primary thrombolysis |
| Shukla et al. [11] | 2018 | Case report | 1 | 57 | Primary thrombolysis |
| Wheatley et al. [27] | 1996 | Case series | 4 | 44.8 | Primary thrombolysis of palmar and digital thrombosis |
| Wörnle et al. [28] | 2004 | Case report | 1 | 69 | Primary thrombolysis |
| Yakubov et al. [29] | 1993 | Case report | 1 | 49 | Primary thrombolysis |
| Zayed et al. [10] | 2013 | Case report | 1 | 26 | Primary thrombolysis |

‡: actual patient age is listed if number of patients n=1. Mean age is listed if n>1.
